# The Cannabinoid Receptor Type 1 Is Essential for Mesenchymal Stem Cell Survival and Differentiation: Implications for Bone Health

**DOI:** 10.1155/2013/796715

**Published:** 2013-06-24

**Authors:** Aoife Gowran, Katey McKayed, Veronica A. Campbell

**Affiliations:** ^1^Discipline of Physiology, School of Medicine, Trinity Biomedical Sciences Institute, University of Dublin, Trinity College, Dublin 2, Ireland; ^2^School of Engineering, Trinity Centre for Bioengineering, Trinity Biomedical Sciences Institute, University of Dublin, Trinity College, Dublin 2, Ireland

## Abstract

Significant loss of bone due to trauma, underlying metabolic disease, or lack of repair due to old age surpasses the body's endogenous bone repair mechanisms. Mesenchymal stem cells (MSCs) are adult stem cells which may represent an ideal cell type for use in cell-based tissue engineered bone regeneration strategies. The body's endocannabinoid system has been identified as a central regulator of bone metabolism. The aim of the study was to elucidate the role of the cannabinoid receptor type 1 in the differentiation and survival of MSCs. We show that the cannabinoid receptor type 1 has a prosurvival function during acute cell stress. Additionally, we show that the phytocannabinoid, Δ^9^-Tetrahydrocannabinol, has a negative impact on MSC survival and osteogenesis. Overall, these results show the potential for the modulation of the cannabinoid system in cell-based tissue engineered bone regeneration strategies whilst highlighting cannabis use as a potential cause for concern in the management of orthopaedic patients.

## 1. Introduction

Mesenchymal stem cells (MSCs) are multipotent adult stem cells present in the bone marrow which can differentiate along several lineages, for example, bone, cartilage, and tendon [1]. Musculoskeletal repair relies on a series of orchestrated events that direct the differentiation of MSCs to its progeny, for example, osteoblasts, chondrocytes, and tenocytes. MSCs represent an ideal cell population for use in tissue engineering and regenerative medicine due to their ease of isolation, multipotency, lack of immunogenicity, and immunosuppressive effects [2]. Tissue engineering aims to learn how to induce, modulate and control the differentiation process of MSCs in order to provide therapeutics for musculoskeletal diseases [3]. We have recently shown that the osteogenic and chondrogenic differentiation process may be controlled by specific growth factors [4], hypoxia [5], and biophysical stimulation [6]. 

The endocannabinoid system is comprised of two G protein-coupled receptors, CB_1_ and CB_2_, the endogenous ligands anandamide and 2-arachidonoylglycerol, and their degradative enzymes fatty acid amide hydrolase and monoacylglycerol lipase, respectively. In addition, exogenous cannabinoids such as the bioactive lipids isolated from the *Cannabis sativa *plant and synthetic cannabinoids are currently used therapeutically for a number of diseases such as multiple sclerosis [7]. However, phytocannabinoids have a dual toxicity profile with the psychoactive component of cannabis, Δ^9^-Tetrahydrocannabinol (Δ^9^-THC), inducing cell death in a number of cell types [8–11]. Δ^9^-THC is a partial agonist of the CB_1_ and CB_2_ receptors but displays higher efficacy at CB_1_ over CB_2_ where it has reported antagonist activity [12].

The endocannabinoid system is an important regulator of bone mass maintenance. In 2005, Idris et al. reported that CB_1_ receptor inactivation resulted in increased bone mass and protected against ovariectomy-induced bone loss, an *in vivo *model of osteoporosis [13]. Further investigation of the skeletal phenotype of CB_1_ knock-out mice has demonstrated that animals display increased bone mass at 3 months of age, due to reduced osteoclast activity, but develop age-related osteoporosis by 12 months, due to enhanced adipocyte differentiation [14]. CB_2_ receptor agonists increase bone mass by enhancing osteoblast numbers and activity, inhibiting the proliferation of osteoclasts and stimulating fibroblastic colony formation by bone marrow cells [15, 16]. Furthermore, CB_2_ regulates bone loss during periods of increased bone turnover also involving the regulation of osteoclast function [17].

The aim of the present study was to elucidate the role of the cannabinoid system in the survival and differentiation of culture-expanded MSCs in the presence of known osteogenic factors: dexamethasone, *β*-glycerophosphate, and ascorbic acid. The results demonstrate that the CB_1_ receptor is upregulated during osteogenic differentiation of MSCs and is essential for the survival of differentiated MSCs. We also show that the psychoactive phytocannabinoid, Δ^9^-Tetrahydrocannabinol, has a negative impact on MSC survival and osteogenesis.

## 2. Materials and Methods

### 2.1. Culture of Mesenchymal Stem Cells

Three-month-old Wistar rats (250–300 g) were obtained from the Bioresources Unit, University of Dublin, Trinity College. Animals were sacrificed by CO_2_ asphyxiation and cervical dislocation in accordance with European guidelines (86/609/EEC). The femur and tibia were dissected free and placed in sterile prewarmed supplemented Dulbecco's modified Eagle's medium (s-DMEM; Sigma-Aldrich, UK). Supplements were 10% foetal bovine serum; 100 U/mL penicillin/streptomycin; 2 mM GlutaMAX; 1 mM L-glutamine; and 1% nonessential amino acids (Invitrogen, Scotland). The femur and tibia were cut at both epiphyses, and bone marrow was flushed into a 50 mL tube using 5 mL s-DMEM and a 25-gauge needle. The suspension was centrifuged (650 × g) for 5 minutes at 20°C, resuspended in 10 mL of s-DMEM, and passed sequentially through 16-, 18-, and 20-gauge needles. The suspension was passed through a 40 *μ*m nylon mesh into a sterile Petri dish and incubated in a humidified atmosphere (95% air and 5% CO_2_) at 37°C for 30 min. The supernatant was removed and split between two T75 flasks. Culture media was replaced following 24 hours to remove nonadherent cells. Cells were passaged upon reaching 80–90% confluency to a maximum of 4 passages. The medium was replaced every 3 to 4 days. To induce osteogenesis, cells were treated with osteogenic factors (OF): 100 nM dexamethasone, 10 mM *β*-glycerophosphate and 50 *μ*M ascorbic acid for the indicated time period (2–5 weeks). These cells are referred to as differentiated cells, whilst cells maintained in regular culture medium are referred to as undifferentiated cells. Additionally, the differentiation capacity of MSCs was investigated and verified using previously described methods for the induction and detection of osteogenesis, chondrogenesis [4], and adipogenesis [18] in bone marrow derived MSCs (see Figure 1 in Supplementary Material available online at http://dx.doi.org/10.1155/2013/796715). 

### 2.2. Drug Treatments

MSCs were incubated with drugs or vehicle for the time indicated in each experiment. The CB_1_ receptor antagonist/inverse agonist SR141716 was a kind gift form Dr. David Finn at The National University of Ireland, Galway (original source: The National Institute of Mental Health's Chemical Synthesis and Drug Supply Program). SR141716 was stored as a 10 mM stock solution in DMSO at −20°C and diluted to a final concentration of 1 *μ*M in culture media. Δ^9^-THC was obtained from Sigma-Aldrich Company Ltd. and held under license granted by the Irish Department of Health and Children. Δ^9^-THC was stored as a 80 mM stock solution in ethanol at −20°C and diluted to a final concentration of 1 *μ*M in culture media. 

### 2.3. RNA Isolation

Total RNA was isolated from MSCs using a NucleoSpin total RNA isolation kit (Macherey-Nagel Inc., Germany) following the manufacturer's instructions. This protocol included a DNase step in order to remove any genomic DNA contamination. Total RNA concentrations were determined by spectrophotometry (NanoDrop Technologies, USA) and stored at −80°C until required for cDNA synthesis. 

### 2.4. cDNA Synthesis

Total RNA concentrations were adjusted to a standard concentration prior to cDNA synthesis. cDNA was generated from 0.5–1 *μ*g total RNA using High Capacity cDNA Archive kit (Applied Biosystems, Germany) following the manufacturer's instructions. The resultant cDNA was stored at −20°C until required for real time PCR.

### 2.5. Real-Time PCR

Real-time PCR was performed using Taqman Gene Expression Assays (Applied Biosystems, Germany) on an ABI Prism 7300 instrument (Applied Biosystems, Germany). The assay IDs for the genes examined were as follows: CB_1_ receptor (Rn00562880_m1), CB_2_ receptor (Rn01637601_m1), osteocalcin (Rn00566386_g1), and *β*-actin (4352340E). Gene expression was calculated relative to the endogenous control (*β*-actin) and to the control samples to give a relative quantification (RQ) value. 

### 2.6. Cell Viability Assay

Cell viability was determined by quantifying the enzymatic conversion of cell permeable calcein AM (Invitrogen, Scotland) to a fluorescent product by active intracellular esterases. Briefly, MSCs were grown on sterile 96 well plates (6 × 10^3^ cells per well) and treated as indicated in each experiment. Calcein AM solution (2 *μ*M in PBS) was applied to each well and incubated in a humidified atmosphere (95% air and 5% CO_2_) at 37°C for 1 hour. Following incubation calcein fluorescence at 530 nm was determined using a microplate reader heated to 37°C (Synergy HT, BioTek Instruments, USA).

### 2.7. Immunofluorescent Staining for Active Caspase-3 and Apoptotic Nuclei Determination

Following drug treatment, MSCs were fixed in 100% methanol for 5 minutes at −20°C, permeabilised with 0.2% Triton-X100 for 10 minutes, and washed in 3 changes of PBS at room temperature (RT). MSCs were blocked with 30% goat serum overnight at 4°C (Vector Laboratories, USA). Caspase-3 was labelled with a rabbit antiactive caspase-3 (1 : 1000 in 30% blocking buffer; Promega, England) for 1 hour at RT. Labelled protein was detected with goat anti-rabbit secondary antibody conjugated to biotin (1 : 1500 in 30% blocking buffer; Vector Laboratories, USA) for 1 hour at RT. MSCs were then incubated with avidin-conjugated FITC (1 : 500; Sigma-Aldrich, England) for 1 hour at RT. Nuclei were stained with Hoechst 33258 (1 : 500; Invitrogen, Scotland) for 15 minutes at RT. Coverslips were mounted with mounting medium (Vector Laboratories, USA). Incorporated fluorophores were examined with a confocal microscope (Carl Zeiss, Germany) using appropriate excitation wavelengths and filter sets. The number of abnormal apoptotic nuclei was determined (by a blinded counter) from 10 random fields of view for each treatment group with the *n* number indicated in each experiment. 

### 2.8. Extracellular Matrix Mineralization Quantification

The specific marker of mineralized bone, hydroxyapatite, was quantified using a commercially available assay kit (Lonza, Switzerland) following the manufacturer's instructions. Briefly, MSCs were grown on sterile 96 well plates (13 × 10^3^ cells per well) and treated as indicated in each experiment. Following treatment, MSCs were washed in PBS (×2) and then fixed in 100% ethanol for 20 minutes at RT. MSCs were incubated with fluorescent staining reagent specific for hydroxyapatite for 30 minutes at RT. MSCs were washed in diluted wash buffer (×3), and fluorescence was read at 518 nm using a spectrophotometer (Labsystems, Finland). In some experiments, MSCs were grown on glass coverslips and stained with the fluorescent staining reagent specific for hydroxyapatite. Nuclei were stained with Hoechst 33258. Labelled hydroxyapatite and nuclei were visualized with a confocal microscope (Carl Zeiss, Germany) using appropriate excitation wavelengths and filter sets.

### 2.9. Statistical Analysis

Data are reported as the mean ± SEM of the number of experiments indicated in each case. ANOVA followed by a Student Newman-Keuls *post hoc* test was used to determine the statistical significance between groups. For comparisons between relevant treatments, an unpaired Student's *t-*test was performed. 

## 3. Results

### 3.1. Increased CB_1_ Receptor Expression Is Responsible for MSC Survival during Osteogenesis

**Figure 1:**
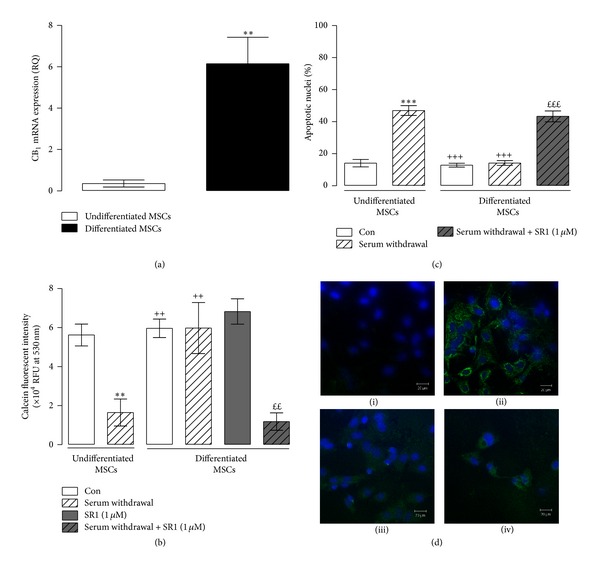
The CB_1_ receptor is increased during early osteogenesis and is essential for the survival of differentiated MSCs. (a) Differentiated MSCs displayed a significant increase in CB_1_ receptor mRNA expression after 2 weeks of differentiation compared to undifferentiated MSCs (**P = 0.002, Student's unpaired *t*-test, *n* = 5). (b) Serum withdrawal significantly reduced the metabolic function of undifferentiated MSCs (Con; **P < 0.01, 1-way ANOVA and Newman-Keuls, *n* = 5). In differentiated MSCs, serum withdrawal had no effect on metabolic function, and serum deprived differentiated MSCs displayed significantly greater metabolic function compared to serum deprived undifferentiated MSCs (^++^ *P* < 0.01, 1-way ANOVA and Newman-Keuls, *n* = 5). Treatment of differentiated MSCs with SR141716 (SR1, 1 *μ*M; 24 hours) blocked the ability of differentiated MSCs to survive serum withdrawal (^*££*^ *P* < 0.01, 1-way ANOVA and Newman-Keuls, *n* = 5). (c) Serum withdrawal induced a significant increase in the numbers of undifferentiated MSCs displaying apoptotic nuclei (Con; ***P < 0.001, 1-way ANOVA and Newman-Keuls, *n* = 4) compared to undifferentiated MSCs maintained with serum. Differentiated MSCs survived serum withdrawal compared to serum deprived undifferentiated MSCs (^+++^ *P* < 0.001, 1-way ANOVA and Newman-Keuls, *n* = 4). Treatment of differentiated MSCs with SR1 blocked the ability of differentiated MSCs to survive serum withdrawal compared to serum deprived differentiated MSCs (^*£££*^ *P* < 0.001, 1-way ANOVA and Newman-Keuls, *n* = 4). (d) Representative images of caspase-3 activity in undifferentiated MSCs exposed to control (i) and (ii) serum withdrawal conditions and caspase-3 activity in differentiated MSCs exposed to control (iii) and serum withdrawal in the presence of SR1 (iv).

As MSCs underwent osteogenic differentiation, a significant increase in CB_1_ receptor mRNA expression was observed after 2 weeks of differentiation (6.15 ± 1.28; RQ value, mean ± SEM) compared to undifferentiated MSCs (0.36 ± 0.17; RQ value, mean ± SEM; *P* = 0.002, Student's unpaired *t*-test, *n* = 5; Figure 1(a)). No change in CB_2_ receptor mRNA expression was observed between undifferentiated and differentiated MSCs (supplemental Figure 2). 

Since an induction of CB_1_ receptor mRNA was evident in MSCs undergoing osteogenic differentiation, we sought to identify whether the induction of the CB_1_ receptor was pertinent in the control of any aspect of MSC function and focused our attention on cell survival. Undifferentiated and differentiated MSCs were deprived of serum in the presence or absence of the CB_1_ receptor antagonist/inverse agonist, SR141716 (SR1; 1 *μ*M), and cell viability was measured by monitoring the metabolism of calcein AM. In undifferentiated MSCs, fluorescent intensity at 530 nm, a marker of cellular metabolism and viability, was 5.62 ± 0.56 (×10^4^ RFU at 530 nm, mean ± SEM), and this was significantly reduced to 1.6 ± 0.69 following serum withdrawal for 24 hours (*P* < 0.001, 1-way ANOVA and Newman-Keuls, *n* = 5; Figure 1(b)). In contrast, when differentiated MSCs were exposed to serum withdrawal fluorescence was unaffected, indicating that the differentiated MSCs were able to withstand serum withdrawal. However, in the presence of SR141716 (SR1; 1 *μ*M; 24 hours) the differentiated MSCs were unable to survive following serum withdrawal indicating that the increased levels of CB_1_ receptor present in differentiated MSCs are essential for survival. Treatment of differentiated MSCs with SR1 alone had no effect on MSC cell viability indicating that SR1 treatment was not toxic.

In addition, we monitored cell death by assessing the percentage of apoptotic nuclei and the expression of the active form of the proapoptotic protein, caspase-3 (Figures 1(c) and 1(d)). In undifferentiated MSCs, serum withdrawal significantly increased the percentage of apoptotic nuclei from 14 ± 2% to 47 ± 3% (mean ± SEM; *P* < 0.001, 1-way ANOVA and Newman-Keuls, *n* = 4; Figure 1(c)) and also increased the expression of active caspase-3 (Figure 1(d)(ii)). However, in differentiated MSCs serum withdrawal evoked significantly less apoptosis (14 ± 1% apoptotic nuclei, mean ± SEM; *P* < 0.001, 1-way ANOVA and Newman-Keuls, *n* = 4; Figure 1(c)). In the presence of SR141716 the apoptotic effect of serum withdrawal was restored (43 ± 3% apoptotic nuclei) in the differentiated MSCs. These results provide evidence that the CB_1_ receptor in differentiated MSCs is essential for survival following an insult such as serum withdrawal. 

### 3.2. Δ^9^-THC Negatively Impacts on MSC Viability and Osteogenic Potential

Given that we have shown an essential role for the CB_1_ receptor in the survival of MSCs during stressful stimulus (serum withdrawal) we therefore sought to elucidate if exogenous cannabinoids could interfere with MSC viability and differentiation capacity. Hence, we monitored the effect of exogenous phytocannabinoid Δ^9^-THC on the viability and osteogenic capacity of MSCs. 

**Figure 2:**
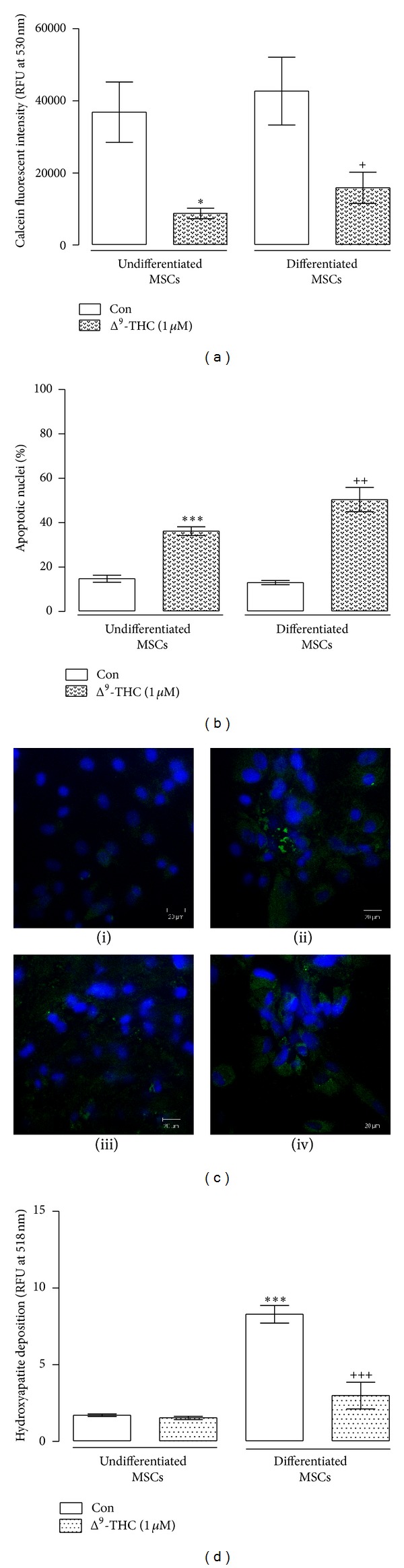
Δ^9^-THC negatively affects MSC viability and inhibits MSC osteogenesis. (a) Treatment of undifferentiated MSCs with Δ^9^-THC (1 *μ*M) significantly reduced viability compared to control undifferentiated MSCs (Con; *P < 0.05, 1-way ANOVA and Newman-Keuls, *n* = 5). Also, differentiation of MSCs in the presence of Δ^9^-THC significantly decreased viability compared to control differentiated MSCs (Con; ^+^ *P* < 0.05, 1-way ANOVA and Newman-Keuls, *n* = 5). (b) Treatment of undifferentiated MSCs with Δ^9^-THC induced a significant increase in the percentage of apoptotic nuclei compared to control MSCs (Con; ***P < 0.001, 1-way ANOVA and Newman-Keuls, *n* = 6). Also, differentiation of MSCs in the presence of Δ^9^-THC significantly increased the percentage of apoptotic nuclei compared to control differentiated MSCs (^++^ *P* < 0.001, 1-way ANOVA and Newman-Keuls, *n* = 6). (c) Representative images of cells stained for active caspase-3 in control undifferentiated MSCs (i), undifferentiated MSCs treated with Δ^9^-THC (ii), control differentiated MSCs (iii), and differentiated MSCs in the presence of Δ^9^-THC (iv). (d) Differentiation of MSCs in the presence of Δ^9^-THC (1 *μ*M) significantly decreased hydroxyapatite deposits compared to control differentiated MSCs (^+++^ *P* < 0.001, 1-way ANOVA and Newman-Keuls, *n* = 6).

The effect of the Δ^9^-THC on the viability of MSCs was determined by assessing the ability of undifferentiated and differentiated MSCs treated with Δ^9^-THC to metabolise calcein AM. Treatment with Δ^9^-THC (1 *μ*M, 2 weeks) significantly reduced undifferentiated MSC metabolic activity from 3.68 ± 0.83 (×10^4^ RFU at 530 nm, mean ± SEM) to 0.88 ± 0.15 (*P* < 0.05, 1-way ANOVA and Newman-Keuls, *n* = 5; Figure 2(a)). In differentiated MSCs treatment with Δ^9^-THC (1 *μ*M, 2 weeks) induced a significant decrease in MSC metabolic activity (*P* < 0.05, 1-way ANOVA and Newman-Keuls, *n* = 5; Figure 2(a)). Additionally, treatment of undifferentiated and differentiated MSCs with Δ^9^-THC (1 *μ*M, 2 weeks) evoked a significant increase in the % of apoptotic nuclei (*P* < 0.001, 1-way ANOVA and Newman-Keuls, *n* = 6; Figure 2(b)) and caspase-3 activity (Figure 2(c)). 

The effect of the Δ^9^-THC on the differentiation of MSCs was determined by monitoring hydroxyapatite deposits in undifferentiated and differentiated MSCs. Deposits of hydroxyapatite were significantly increased from 1.71 ± 0.07 (RFU at 518 nm, mean ± SEM) to 8.30 ± 0.57 in MSCs differentiated with OF (*P* < 0.001, 1-way ANOVA and Newman-Keuls, *n* = 6; Figure 2(d)). However, MSCs differentiated with OF in the presence of Δ^9^-THC had reduced osteogenic potential (2.30 ± 0.87, RFU at 518 nm, mean ± SEM; *P* < 0.001 1-way ANOVA and Newman-Keuls, *n* = 6; Figure 2(d)). These results indicate that the phytocannabinoid Δ^9^-THC has a negative effect on osteogenesis by decreasing the survival of both undifferentiated and differentiated MSCs.

## 4. Discussion

The aim of this study was to examine the role of the CB_1_ receptor during the osteogenic differentiation of MSCs harvested from adult Wistar rats. The results demonstrate that the CB_1_ receptor is increased during MSC osteogenic differentiation and is essential for the survival of differentiated MSCs during the acute insult of serum withdrawal. We also show that the exogenous phytocannabinoid, Δ^9^-THC, reduced MSC survival and differentiation potential of MSCs. 

Substantial loss of bone due to trauma, tumour ressection, metabolic bone disease or lack of bone repair due to ageing may require intervention to restore a positive balance to bone metabolism [19]. MSCs represent an ideal adult stem cell for the use in bone repair since strategies for bone regeneration (osteogenesis, osteoinduction, osteoconduction, and osteopromotion) all fundamentally rely on MSCs [20]. We have observed that MSCs produce osteocalcin and extracellular hydroxyapatite deposits (supplemental Figures  1 and 3) confirming the potential of isolated MSCs to become bone forming cells suitable for use in bone tissue engineering strategies in accordance with previously established criteria [21, 22]. The CB_1_ and CB_2_ receptors are G-protein coupled receptors which are currently being assessed, along with the putative cannabinoid receptor GPR55, as potential modulators of bone mass [23, 24]. It has been previously established that MSCs express CB_1_ receptors [12, 14, 15], however, we are the first to show a functional increase of the CB_1_ receptor during osteogenesis. We did not observe any increase in CB_2_ receptor expression (supplemental Figure  2); however, this may be due to the time point analysed (2 weeks) as expression of the CB_2_ receptor has previously been found to be expressed after 3 weeks of osteogenic differentiation in murine bone marrow-derived primary stromal cells [15]. We have also shown that the CB_1_ receptor has a functional role in the survival of differentiated MSCs exposed to an acute insult (serum withdrawal), which is an *in vitro* model of the environment surrounding bone fractures or orthopaedic implants. Our results indicate that the CB_1_ receptor is required for MSC survival during the early stages of MSC osteogenesis. Successful fracture repair and bone healing around orthopaedic implants rely on favourable biological and mechanical environments in addition to the recruitment and differentiation of MSCs. However, in certain circumstances the local environment can be actively inhospitable to infiltrating MSCs resulting in the failure of bone healing [20, 25]. The CB_1_ receptor has been demonstrated to be cytoprotective in many cell types [26, 27]. In our study we show that differentiated MSCs have increased CB_1_ receptor and display the ability to survive an acute insult (serum withdrawal) compared to undifferentiated MSCs. Interestingly, Cudaback and coworkers [28] have demonstrated that increased cannabinoid receptor expression changes the coupling of these receptors to specific kinase pathways and the efficacy by which cannabinoid receptor ligands induce the activation of these pathways. Furthermore, they showed that increased CB_1_ receptor expression enhanced the efficacy of cannabinoids to regulate the prosurvival AKT pathway whilst low levels of CB_1_ receptor expression lead only to the activation of ERK [28]. Furthermore, we have previously shown that activation of the cannabinoid system enhances the survival, migration, and chondrogenic differentiation of MSCs, which are the three key points that determine the success of cell-based tissue-engineered repair strategies [29]. Interestingly, Idris et al. [13] suggest that normal bone formation in CB_1_ receptor knock-out mice can be maintained by alternative signalling pathways; however, with increasing age these compensatory mechanisms fail leading to decreased bone formation. Furthermore, the physiological upregulation of the CB_1_ receptor with age has been proposed to protect against the development of osteoporosis [13]. Results from our experiments using SR141716 show that the CB_1_ receptor is necessary for MSC survival following an acute insult, yet long term (3–5 weeks) CB_1_ receptor antagonism results in increased osteogenesis (supplemental Figure  3) indicating a temporal effect of the CB_1_ receptor on MSC function. This novel temporal response may reflect a dual role for the CB_1_ receptor in MSC physiology: firstly being essential for survival during stress which is of relevance to the inhospitable environment present around areas of bone healing and secondly acting as a brake on osteogenesis, reflective of endocannabinoids having an inhibitory role during osteogenesis. The osteogenic effect of long-term CB_1_ receptor antagonism that we observed may be due to enhanced signalling through the CB_2_ receptor, since CB_2_ receptor signalling leads to expansion of the preosteoblastic pool and increased numbers of osteoblastic colony formation [14, 15]. Furthermore, CB_2_ receptor activation attenuates bone loss in an animal model of bone cancer metastases using sarcoma cells [30]. Further studies utilizing CB_1_ and CB_2_ knock-out animals will be necessary to dissect out the exact role of both receptors and to corroborate our findings. Alternatively SR141716 may be signalling through other receptors such as PPAR-*γ* [31].

Our results also demonstrate that Δ^9^-THC prevents osteogenesis and induces cell death in both undifferentiated and differentiated MSCs. These findings may provide a molecular explanation for the results of Nogueira-Filho and coworkers [32] who showed reduced cancellous bone healing around titanium implants, due to a reduction in bone filling in rats subjected to cannabis smoke inhalation. In contrast, the nonpsychoactive component of cannabis, cannabidiol has been shown to reduce bone resorption during experimental periodontitis in rats due to the reduction in proinflammatory mediators [33]. It has been reported that Δ^9^-THC is a mitochondrial inhibitor [34], an effect that may inhibit the survival of MSCs and osteoblasts since mitochondrial function determines the viability and osteogenic potency of these cells [35]. These reports further emphasise the relevance of our observations that Δ^9^-THC exposure increases numbers of apoptotic nuclei and induced the expression of active caspase-3 (a proapoptotic downstream signalling protease involved in the mitochondrial intrinsic pathway of apoptosis) in undifferentiated and differentiated MSCs. Thus, Δ^9^-THC exposure may lead to a decreased ability of MSCs to differentiate into their mature bone forming progeny due to a lack of cell viability at early stages of osteogenesis which could inturn impact upon the osteogenic potential of MSCs (supplemental Figure  4). Furthermore we conclude that this effect is specific to a long-term treatment with Δ^9^-THC as we have previously published observations showing no deleterious effects following an acute 24-hour Δ^9^-THC (1 *μ*M) treatment [29]. This indicates that a long-term exposure to Δ^9^-THC may have a negative effect on bone health possibly due to exogenous agonist-induced blockade of CB_1_ receptor activation by endocannabinoids. However, further studies need to be carried out to confirm this. These results have important clinical implications for bone repair in cannabis users or self-medicating orthopaedic patients since it has already been clearly established that tobacco and alcohol consumption negatively impacts on bone health [36].

In summary, we have obtained additional insights into the role of the cannabinoid system in the regulation of bone maintenance by investigating the cannabinoid system during MSC osteogenic differentiation. Herein we show that the CB_1_ receptor is induced during osteogenic differentiation and that it has a functional role in MSC survival during acute stress. These results are relevant to the successful culturing of osteogenic progenitor cells used in cell-based tissue engineered bone replacement therapies as a cannabinoid based approach may overcome the challenges associated with cell senescence and donor site morbidity present in current tissue engineered applications. Indeed, the concept of priming cells with specific growth factors or receptor specific ligands has been shown to control the differentiation potential and immunomodulatory profile of MSCs [37, 38]. In view of this, our results demonstrate the potential application of cannabinoids to prime MSCs in order to influence their *in vitro* and *in vivo* physiological functions representing an intriguing avenue for further research. We also provide evidence that the phytocannabinoid Δ^9^-THC has a negative impact on MSC osteogenesis and survival. This may be a relevant factor which should be considered as a potential source of risk in the rate of clinical success of any bone replacement strategies. 

## Supplementary Material

Supp. Fig. 1: The differentiation capacity of MSCs.
Supp. Fig. 2: The effect of osteogenic factors on CB2 receptor expression.
Supp. Fig. 3: The effect of CB1 receptor antagonism on MSC osteogenesis.
Supp. Fig. 4: The effect of ∆9-THC on osteocalcin and CB1 receptor mRNA expression.

## Abbreviations

MSCs: Mesenchymal stem cells
OF: Osteogenic factors
CB: Cannabinoid
SW: Serum withdrawal.
