# Supplementary material for: The Cannabinoid Receptor Type 1 Is Essential for Mesenchymal Stem Cell Survival and Differentiation: Implications for Bone Health
Source: Stem Cells Int. 2013 Jun 24;2013:796715. doi: 10.1155/2013/796715 (PMC3707275; doi:10.1155/2013/796715)
Supplement: Supplementary file 1 — Supp. Fig. 1: The differentiation capacity of MSCs. Supp. Fig. 2: The effect of osteogenic factors on CB2 receptor expression. Supp. Fig. 3: The effect of CB1 receptor antagonism on MSC osteogenesis. Supp. Fig. 4: The effect of ∆9-THC on osteocalcin and CB1 receptor mRNA expression. [file 796715.f1.doc]

**Supplemental Figures**

**Supp. Fig. 1: The differentiation capacity of MSCs.**

Representative images showing MSCs treated with osteogenic factors for 3 weeks (ii) displayed increased extracellular deposits of hydroxyapatite compared to control MSCs (i). MSCs treated with adipogenic factors for 2 weeks (iv) displayed enahced lipid deposits indicative of adipogenesis compared to untreated MSCs (iii). MSCs treated with chondrogenic factors for 3 weeks (vi) exhibited altered morphology and increased proteoglycan expression compared to control MSCs (v).

**Supp. Fig. 2: The effect of osteogenic factors on CB_2_ receptor expression.**

No significant change in CB_2_ receptor mRNA expression was observed between undifferentiated and differentiated MSCs, although CB_2_ receptor mRNA was highly expressed in undifferentiated MSCs.

**Supp. Fig. 3: The effect of CB_1_ receptor antagonism on MSC osteogenesis.**

A, osteogenic differentiation for 2 weeks induced a significant increase in osteocalcin mRNA expression compared to control undifferentiated MSCs (Con; ****p*<0.001, 1-way ANOVA and Newman-Keuls, *n*=3).

B, osteogenic differentiation for 5 weeks induced a significant increase in hydroxyapatite deposits in the extracellular matrix compared to control undifferentiated MSCs (Con; ****p*<0.001, 1-way ANOVA and Newman-Keuls, *n*=6). Differentiation of MSCs in the presence of SR1 (1μM) significantly increased hydroxyapatite deposits compared to control differentiated MSCs (+++*p*<0.01, 1-way ANOVA and Newman-Keuls, *n*=6).

**Supp. Fig. 4: The effect of ∆^9^-THC on osteocalcin and CB_1_ receptor mRNA expression.**

A, treatment of undifferentiated and differentiated MSCs with ∆^9^-THC (1μM) had no significant effect on osteocalcin mRNA expression.

B, treatment of undifferentiated and differentiated MSCs with ∆^9^-THC (1μM) had no significant effect on CB_1_ receptor mRNA expression.


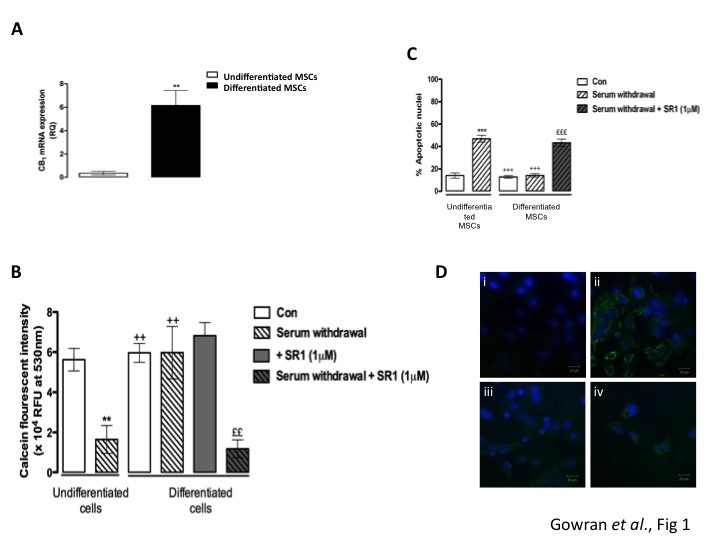


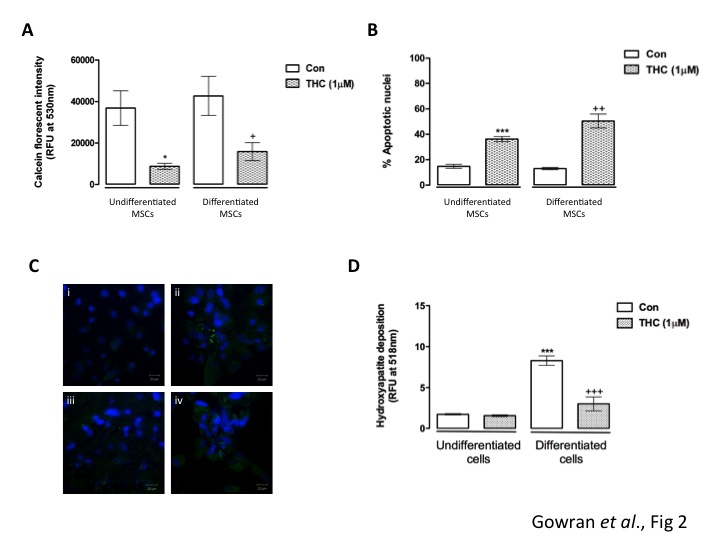


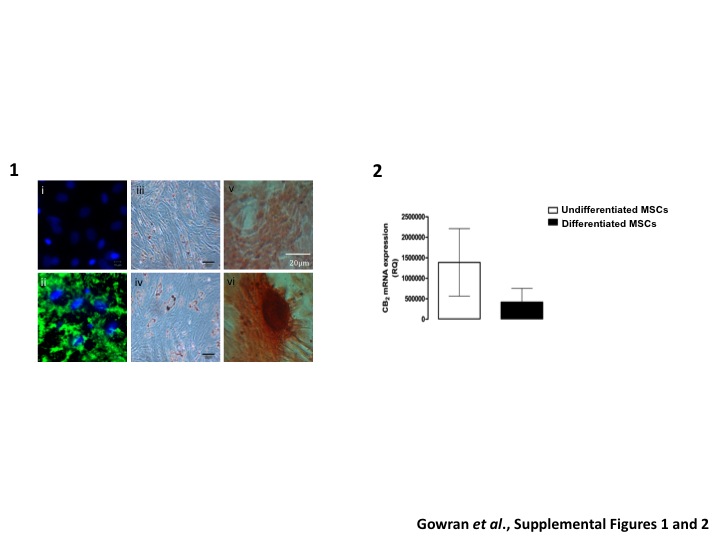


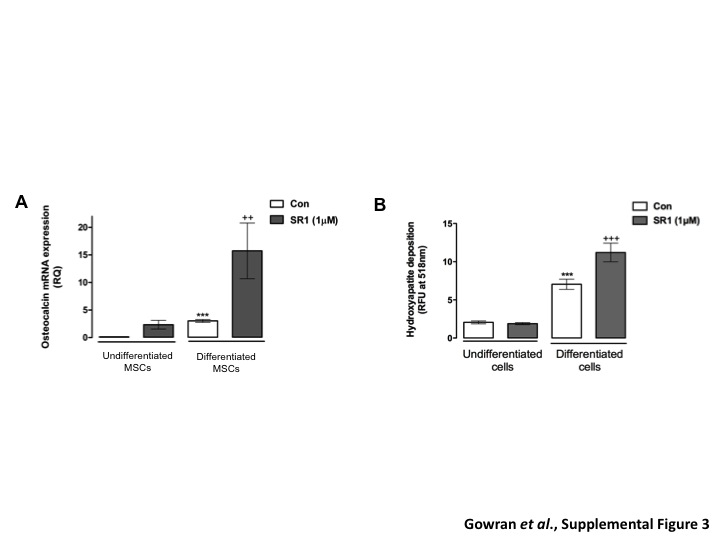


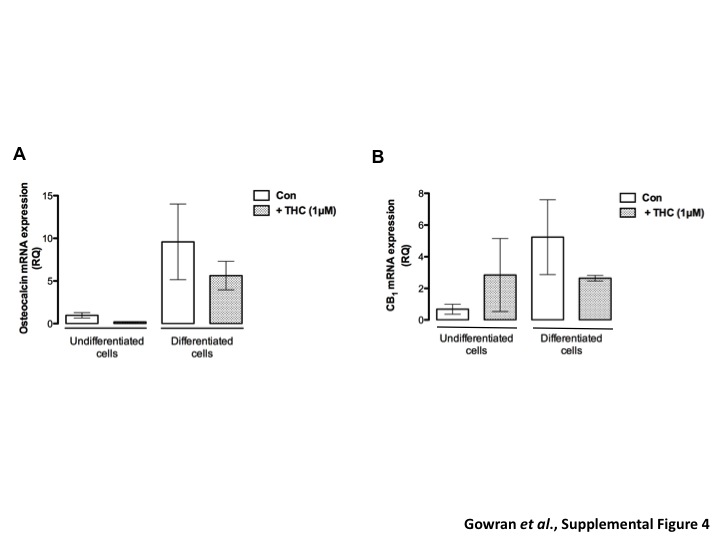


**Supplemental Material Summary**

Bone marrow derived culture expanded MSCs showed tri-lineage differentiation potential as assessed by histochemical analysis of hydroxyapatite deposits following treatment with osteoinductive factors (OF; Figure 1, (ii)); lipid deposits following treatment with adipogenic factors (AF; Figure 1, (iv)); and proteoglycan deposits following treatment with chondroinductive factors (CF; Supplemental Figure 1, (vi)).

There was no significant changes in CB_2_ receptor mRNA as detected by qPCR following 2 weeks of osteogenic differentiation (Supplemental Figure 2). CB_2_ mRNA levels were 1.39x10^6^ ± 0.82 (RQ value, mean ± SEM, *n*=4) compared to 0.42 ± 0.34 (RQ value, mean ± SEM, *n*=4) in undifferentiated MSCs.

MSCs were treated with SR141716 (SR1; 1μM) for 2-5 weeks in the presence or absence of osteogenic factors (OF) and osteogenic differentiation was assessed by monitoring osteocalcin mRNA expression and hydroxyapatite deposits in the extracellular matrix (Supplemental Figure 3 A and B). MSCs that were differentiated in the presence of SR141716 displayed significantly increased osteocalcin mRNA expression (*p*<0.01, 1-way ANOVA and Newman-Keuls, *n*=3; Supplemental Figure 3A). Furthermore, MSCs differentiated in the presence of SR141716 also had significantly increased mineralization of the extracellular matrix (*p*<0.01, 1-way ANOVA and Newman-Keuls, *n*=6; Supplemental Figure 3 B).

MSCs were treated with Δ^9^-THC (1μM) for 2 weeks in the presence or absence of osteogenic factors (OF) followed by the determination of osteocalcin and CB_1_ receptor mRNA levels by qPCR (Supplemental Figure 4 A and B). Overall there was no statistically relevant results for osteocalcin and CB_1_ receptor mRNA levels in undifferentiated or differentiated MSCs.

I have a few minor edits that I wish to include:

Pg 5 line 14 (Figures 2(c) and (d)) should be changed to (Figures 1(c) and (d))

Pg 5 line 23 (Figure 2(c)) should be changed to (Figure 1(c))

Pg 7 line 11 (supplemental Figure 3) should be changed to (supplemental Figure 4)
